# Topologies of a Substrate Protein Bound to the Chaperonin GroEL

**DOI:** 10.1016/j.molcel.2007.04.004

**Published:** 2007-05-11

**Authors:** Nadav Elad, George W. Farr, Daniel K. Clare, Elena V. Orlova, Arthur L. Horwich, Helen R. Saibil

**Affiliations:** 1 Department of Crystallography, Birkbeck College London, Malet Street, London WC1E 7HX, UK; 2 Department of Genetics, Yale University School of Medicine, Boyer Center, 295 Congress Avenue, New Haven, CT 06510, USA; 3 Howard Hughes Medical Institute, Yale University School of Medicine, Boyer Center, 295 Congress Avenue, New Haven, CT 06510, USA; 4 Department of Molecular Biology, The Scripps Research Institute, 10550 North Torrey Pines Road, La Jolla, CA 92037, USA

**Keywords:** PROTEINS

## Abstract

The chaperonin GroEL assists polypeptide folding through sequential steps of binding nonnative protein in the central cavity of an open ring, via hydrophobic surfaces of its apical domains, followed by encapsulation in a hydrophilic cavity. To examine the binding state, we have classified a large data set of GroEL binary complexes with nonnative malate dehydrogenase (MDH), imaged by cryo-electron microscopy, to sort them into homogeneous subsets. The resulting electron density maps show MDH associated in several characteristic binding topologies either deep inside the cavity or at its inlet, contacting three to four consecutive GroEL apical domains. Consistent with visualization of bound polypeptide distributed over many parts of the central cavity, disulfide crosslinking could be carried out between a cysteine in a bound substrate protein and cysteines substituted anywhere inside GroEL. Finally, substrate binding induced adjustments in GroEL itself, observed mainly as clustering together of apical domains around sites of substrate binding.

## Introduction

Molecular chaperones assist the folding of cellular proteins by binding them in unfolded or misfolded states through exposed hydrophobic residues, preventing such surfaces from causing multimolecular aggregation (Saibil, 2000; Frydman, 2001; Hartl and Hayer-Hartl, 2002; Deuerling and Bukau, 2004; Bukau et al., 2006). Bound proteins are then productively released, in many cases through the binding of ATP. The action of polypeptide binding by chaperones takes place in a broad variety of physiological settings, and the various chaperones each present a hydrophobic binding surface in a particular geometric context that favors recognition of particular nonnative substrate conformations. For example, bacterial trigger factor binds to ribosomes and forms a hydrophobic cradle structure that directly receives nascent polypeptide chains emerging from the exit tunnel (Ferbitz et al., 2004; Kaiser et al., 2006). Hsp70 class chaperones bind extended hydrophobic segments of polypeptide chain, holding them within a hydrophobic arch (Flynn et al., 1991; Zhu et al., 1996; Rudiger et al., 1997). And the chaperonin ring structures bind collapsed nonnative states in a central cavity via a hydrophobic lining (Sigler et al., 1998; Saibil and Ranson, 2002; Fenton and Horwich, 2003). This latter step of binding is of particular interest because it leads to subsequent folding to the native form after ATP-directed rigid body movements release the substrate into an encapsulated cavity, which becomes hydrophilic at this step (Xu et al., 1997).

Studies over a number of years have indicated that substrate proteins bound to an open ring of the bacterial chaperonin GroEL may in general occupy an unfolded state. Supporting this are observations both that proteins stably bound to GroEL are unstructured and that binding of nonnative protein in an open ring of GroEL may be associated with unfolding. In the former case, a host of hydrogen exchange experiments performed on stable GroEL-substrate complexes reveal little or no stable secondary structure in bound substrate protein (Robinson et al., 1994; Zahn et al., 1994; Goldberg et al., 1997; Chen et al., 2001). In addition, a recent NMR experiment directly examining isotopically labeled human DHFR while bound to GroEL also revealed no stable structure and suggested a degree of ongoing conformational exchange (Horst et al., 2005). Concerning an action of unfolding associated with binding, an early experiment observed a “thermodynamic partitioning” behavior of GroEL, in which it favored binding of a less-folded nonnative state of a mutant RNase T1 over a more-folded state (Walter et al., 1996). An experiment with the small RNase, barnase, revealed that catalytic amounts of GroEL could produce global hydrogen exchange (Zahn et al., 1996). And a recent study employing intramolecular FRET between the N- and C-terminal regions of the substrate protein Rubisco observed that the termini become more distantly separated upon binding of the metastable intermediate to the open ring of GroEL, consistent with an unfolding action (Lin and Rye, 2004).

Given both the putative unfolding action of substrate binding and the productive nature of binary complexes, the issue of what a nonnative polypeptide bound inside the chaperonin cavity “looks like” is of immediate interest. Where in the central cavity does substrate protein reside? How many chaperonin subunits does it contact? Does it reside only at the apical domain level? To date, only limited information is available on these points, coming on one hand from genetic and biochemical studies and on the other from a small number of physical studies. Functional studies have indicated, for example, that the hydrophobic binding surface lies on a tier of apical cavity-facing secondary structures, two α helices (H and I), and an underlying extended segment (Fenton et al., 1994). Alteration of any of the hydrophobic residues to hydrophilic character abolishes polypeptide binding. Consistent with this as a binding surface, cocrystallization of small hydrophobic peptides with GroEL revealed them to bind in an extended conformation in a groove between the H and I helices, forming contacts with the apical hydrophobic side chains (Buckle et al., 1997; Chen and Sigler, 1999), albeit this binding closely resembles that of the binding of the GroES mobile loop to GroEL (Xu et al., 1997; Shewmaker et al., 2001). A broader apical binding surface is indeed indicated by ability of both a mobile loop-derived peptide and a substrate-derived α-helical peptide to simultaneously bind to the apical domain without competing (Ashcroft et al., 2002). In additional studies, examining combinations and permutations within a ring of wild-type (WT) and binding-defective mutant apical domains, it was observed that at least three consecutive WT apical domains must be present in order to efficiently bind such substrate proteins as Rubisco or MDH, implying a requirement for a continuous hydrophobic binding surface (Farr et al., 2000). Multivalency of binding was further supported by the observation that crosslinks could be formed between substrate protein and multiple surrounding apical domains.

In an early cryo-electron microscopy (cryo-EM) study, the subunit of mitochondrial malate dehydrogenase (MDH) in complex with GroEL was visualized as additional density at the opening of the central cavity (Chen et al., 1994). Similarly, small-angle neutron scattering of a GroEL-rhodanese binary complex detected the substrate protruding from the central cavity in the shape of a champagne cork (Thiyagarajan et al., 1996). In a more recent EM study employing 7-fold averaging, GroEL-bound glutamine synthetase was visualized as a cylindrical axial density forming thin connections to the apical domains (Falke et al., 2005). Here we present a direct visualization of the asymmetric complexes formed by several structural states of MDH bound to an open ring of GroEL. The topology of bound MDH provides new insights about the nature of binding of a full-length polypeptide substrate within the chaperonin ring.

## Results

### Substrate Density in a 7-Fold Symmetrized GroEL-MDH Complex

Mitochondrial MDH is a homodimer of 33 kDa subunits whose refolding following dilution from denaturant is dependent on GroEL-GroES. Upon dilution from urea into a buffer containing GroEL, unfolded MDH becomes rapidly bound in stable binary complexes that are productive of native enzyme upon subsequent addition of ATP and GroES. By contrast, dilution of urea-denatured MDH into buffer alone leads to quantitative aggregation. For EM studies of GroEL-MDH binary complexes, we sought to achieve a high occupancy of GroEL with MDH and thus diluted the unfolded MDH to produce a 2.5-fold molar excess of MDH to GroEL 14-mer, removing the nonbound aggregated MDH by centrifugation, before applying the complexes to EM grids.

**Figure 1:**
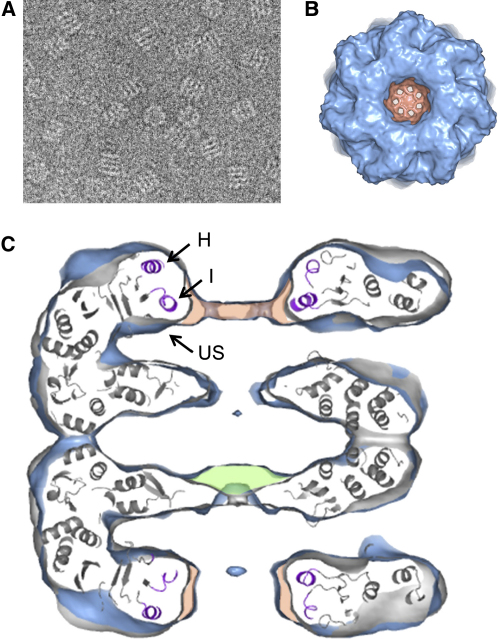
Substrate Density in the Initial 7-Fold Symmetrized GroEL-MDH Complex (A) Representative cryo-EM image of the GroEL(D473C-6His)-MDH complex showing side views (striped rectangles) and end views (rings). (B and C) Structure of the complex made from an initial data set of 8000 images with 7-fold symmetry applied. (B) End view of the GroEL-MDH complex with denatured MDH density colored orange. (C) Central section through the GroEL-MDH cryo-EM map (blue surface) overlaid with the apo GroEL(D473C) 7-fold symmetrized map (gray surface). The two map surfaces coincide almost completely, except in the regions colored orange and green. The atomic structure of GroEL domains fitted to the GroEL-MDH map is shown in gray cartoon format (Braig et al., 1994). Helices H and I and the underlying segment (aa 230–244, 256–271, and 199–203, respectively) in the atomic structure are colored purple and labeled (H, I, US). Extra density in the GroEL-MDH map that can be attributed to nonnative MDH is colored orange. This can be seen mainly adjacent to helix I in the upper ring but also lining the opening of the central cavity of the bottom ring. Extra density corresponding to the C termini, which are more ordered in the GroEL-MDH complex, is colored green. Some additional small displacements of the apical domains are seen in the upper ring.

Examination of the GroEL particles by EM revealed that most of the complexes adopted an end-view orientation, with the 7-fold symmetry axis of GroEL perpendicular to the image plane. This bias to end views is not observed with unbound GroEL, which is distributed in roughly equal proportions between end and side views. Although this difference may indicate good occupancy of MDH in the complexes, 3D reconstruction is not possible with views only along the symmetry axis. Attempts to produce more side views by various pretreatments of the grids were unsuccessful. Complexes with rhodanese, another GroEL substrate, also failed to produce side views. Therefore we resorted to modifying the GroEL molecule itself at the outer surface of the equatorial domains, where a 6-histidine peptide was crosslinked to a cysteine substituted for Asp473. This modification did not alter the ability of GroEL to assist in MDH folding in the presence of GroES and ATP, nor did it alter the structure as seen by cryo-EM (data not shown). When this modified GroEL was complexed with denatured MDH, 50%–80% of the complexes adopted a side-view orientation (Figure 1A).

A 7-fold symmetric reconstruction was first carried out from a data set of 8000 particles (Figures 1B–1D). Processing details are given in Table 1. Atomic coordinates from the GroEL crystal structure (Braig et al., 1994) were docked into the EM density by fitting each of the three GroEL domains individually as rigid bodies, using the program URO (Navaza et al., 2002). Extra density (colored orange) was clearly observed inside the cavity of one GroEL ring (shown as the upper ring in Figure 1B), mainly at the level of helix I, attributed to bound MDH. Additional weaker density appeared also in the opposite ring adjacent to both helix H and I (orange area in lower ring). Extra density (green) is also observed in the regions of the GroEL subunit C termini, which are flexible segments of 20 amino acids that project into the central cavity from the equatorial domains. These segments are not resolved in X-ray structures but are visible in these EM maps as extensions of the equatorial densities into the central cavity of each GroEL ring.

The substrate density in the 7-fold symmetrized map was considerably smaller than expected for a 33 kDa denatured protein. For comparison, the neighboring GroEL apical domains themselves are ∼20 kDa in mass. This partial loss of substrate density is likely due to the application of 7-fold symmetry, which smears out the density of asymmetric structures, and by the variable distribution of substrate in the GroEL cavity. As a result, extra density is observed only on the most highly occupied binding surface, which appears to be the region close to helix I.

### Statistical Analysis of Images Reveals Variations Related to Substrate Occupancy

**Figure 2:**
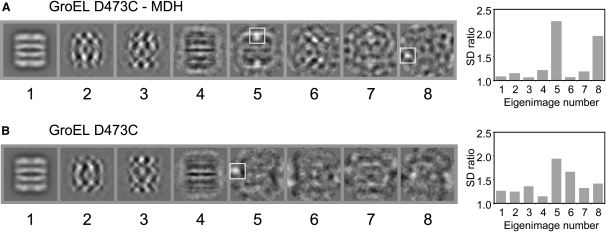
Statistical Analysis of Variations in Substrate Occupancy Eigenimages of (A) initial 8000 images used for the reconstruction of the 7-fold symmetrized GroEL-MDH complex and of (B) 6800 images used for the reconstruction of the 7-fold symmetrized apo GroEL. The ratios between standard deviations of two subregions within the eigenimages are plotted in the corresponding graphs on the right. Two 20 × 20 pixel subregions were selected from each eigenimage, one surrounding the most extreme gray value and the other in the central part of the eigenimage. Subregions with the highest standard deviation are boxed in white (eigenimages A5, A8, and B5). Eigenimages 1–4 are similar in both data sets and are typical of GroEL data sets. The first one corresponds to the average of all images, and 2–4 reflect image variance due to differences in GroEL orientations. In this case, the density variations are evenly distributed over the particle area and there are no prominent local maxima. Eigenimage 5 of the GroEL-MDH data set shows an exceptionally high peak inside one end cavity of GroEL and a similar, somewhat weaker peak in the opposite end cavity. These peaks reflect variation in substrate occupancy, and they do not appear in the corresponding apo GroEL eigenimages. Relatively high peaks appear also in eigenimages of both data sets at the outer surface of the equatorial domains (eigenimages A8 and B5). We attribute this to interactions of Cys473 with other molecules that caused additional independent variations.

The variable substrate occupancy was confirmed by multivariate statistical analysis (MSA), a form of principal component analysis (van Heel and Frank, 1981; van Heel et al., 2000; Frank, 2006) (Figure 2). The purpose of this analysis is to detect significant variations that are likely to be structure related and distinguish them from the finer fluctuations associated with different orientations or noise. The images can then be separated into more homogeneous subsets on the basis of their significant structural variations. Variances in the images are displayed as images in descending order of eigenvalues. These “eigenimages” correspond to the MSA eigenvectors. By using weighting of selected eigenimages, we were able to dissect two major sources of variation in the data set, namely different orientations of the complexes and variable presence of substrate in the GroEL cavity. These two sources of variation are reflected differently in the eigenimages. Orientation differences cause changes throughout the projected density, giving rise to variations distributed evenly over the particle image, typically as stripes or checkerboard features (e.g., Figure 2A, panels 2, 3, and 6), whereas variable substrate occupancy gives rise to local peaks within the cavity regions (e.g., Figure 2A panels 5 and 8). The significance of these latter local variations in the eigenimages can be quantified by measuring the ratios of standard deviation around local peaks to the standard deviation in other regions of the particles (Figure 2, graphs). The eigenimages indicate density variations in the cavity regions of both rings, relating to different locations of substrate (Figure 2A, panel 5). Such variations are not seen in data sets of unbound GroEL(D473C) (Figure 2B) and are distinct from the main variations arising from different orientations of GroEL (Figures 2A and 2B, panels 2–4). The appearance of substantial substrate signals in both rings in the GroEL-MDH data set (Figure 2A, panel 5) is due to mixed up/down orientations of the complexes, because the substrate density did not give a strong enough signal for an accurate alignment at this stage of the processing. Both GroEL-MDH and control data sets show significant peaks at the outer surface of the equatorial domains (Figure 2A, panel 8, and Figure 2B, panel 5), which we attribute to interactions of Cys473 with other molecules.

### Multiple Topologies of Substrate Density Revealed by Classification and Asymmetric Reconstruction

In order to characterize the range of substrate conformations, we classified a data set of 40,000 particles into smaller, more homogeneous subsets on the basis of eigenimages reporting on substrate occupancy and distribution. The classes were progressively subdivided, using substrate-related eigenimages to assess their heterogeneity and further subdivide if possible. Simultaneous projection matching of individual images to multiple models (“competitive” projection matching; see the Experimental Procedures) was used to refine the separation at each stage.

**Figure 3:**
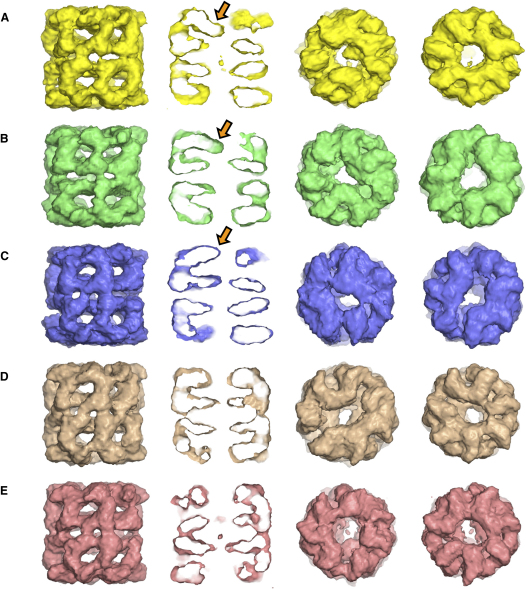
GroEL-MDH Complexes Surface representation of the five different GroEL-MDH asymmetric cryo-EM maps made from the classified images. Shown are side views of the maps in the first column, followed by central sections in the second column, top views in the third column, and bottom views in the fourth column. Classes A–C show significant density for denatured MDH, which is indicated by arrows in the sections and can be seen in the central cavity in the top views.

Ultimately, the data set was separated into five major classes (Figure 3 and Table 1). These showed a high degree of stability in consecutive rounds of competitive alignment and angle assignment. We concluded that this is the maximum number of distinct classes that we can discriminate in this data set. Of the five final classes, three produced structures with well-defined extra densities revealing different positions of nonnative MDH binding (A–C). The other two classes did not exhibit significant extra density in the cavity (D and E). All classes refined to a resolution of 10–11 Å at 0.5 FSC, and sharpened maps show features of the α-helical substructure, as expected in this resolution range (Figure S1 in the Supplemental Data available with this article online, Table 1). Attempts at further classification based on any of the eigenimages that were calculated within classes and showed peaks (Figure S2) did not produce new stable subclasses. These eigenimages largely represent variations arising from orientations of the complexes within the classes, whether that is seen as variation in GroEL orientations around the pseudo 7-fold axis (for example, Figure S2, eigenimages 2 and 3 in all classes) or variation caused by asymmetric density around the central cavity (Figure S2, eigenimage 6 in classes A and B, eigenimage 4 in classes C and D).

**Figure 4:**
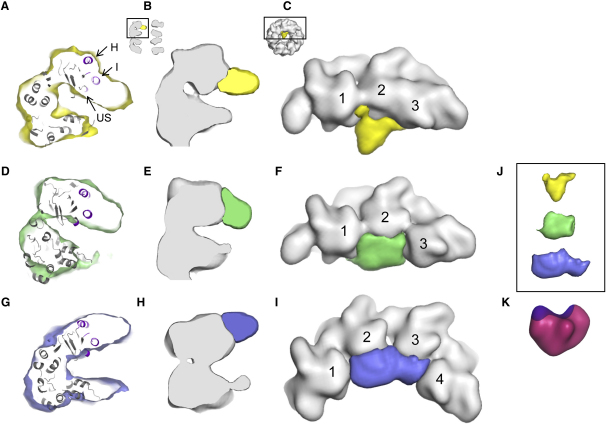
Multiple Topologies of Substrate Density in the Complexes Nonnative MDH density and adjacent GroEL subunits in classes A (A–C), B (D–F), and C (G–I). (A, D, and G) Sections through the substrate-bound subunits in the EM densities (yellow, green, and blue surface representations) with fitted atomic structures of GroEL domains (Braig et al., 1994) (gray cartoon representation). Helices H and I and the underlying segment (aa 230–244, 256–271, and 199–203, respectively) in the atomic structures are colored purple and labeled in (A) (H, I, US). Class A substrate density is located deep inside the GroEL cavity adjacent to helix I and the underlying segment; class B substrate is bound less deeply, mainly to helix I; and class C substrate is located in a more exterior position adjacent to helices H and I. (B, C, E, F, H, and I) Overlay of density derived from the atomic structures, low-pass filtered to 14 Å (gray), with difference maps made by subtracting the filtered fitted model density from the corresponding EM density (colored yellow, green, and blue according to the class). Shown in (B), (E), and (H) are slices through a substrate-bound GroEL subunit and the associated difference density (see inset above [B]). The slices are taken through subunit 2 (C, F, and I). Shown in (C), (F), and (I) are top views of substrate-bound subunits and associated difference density (see inset above [C]). Class A and B substrates associate with three GroEL apical domains, whereas class C substrate associates mainly with four GroEL apical domains. (J) Isolated difference densities showing their variable shapes (yellow, green, and blue). (K) Surface representation of a MDH monomer (Gleason et al., 1994) filtered to 20 Å (magenta) is shown for comparison. The dimer interface of MDH is colored purple.

Classes A and B (together accounting for 36% of images in the final data set) show strong interaction of the substrate with the central α helix of the apical face (helix I) and the underlying segment (aa 199–203) (Figures 3A and 3B and Figures 4A–4C and 4D–4F, respectively). The main substrate density in these two classes is in contact with the apical domains of three consecutive subunits (Figures 4C and 4F). In both cases, the substrate density extends diagonally from the lower part of the apical domains downwards toward the particle axis. Class A substrate density shows the most restricted contact surface with the apical domains, mainly concentrated around helix I and the underlying segment, and it has the smallest observed volume (∼25% of native MDH monomer). The density in class B also contacts helix H (Figure 4D) and stretches horizontally over a wider surface of the three bound apical domains (Figure 4F), with a larger substrate volume observed (∼40% of native MDH monomer). The observed density is apparently an average of flexible MDH monomers within each class, and therefore it reveals only the regions most highly occupied by substrate protein.

Class C contains 13% of the complexes and is distinct from classes A and B described above. The main substrate density is bound in a more external position (∼22 Å higher than the substrate in class A), at the inlet to the central cavity, contacting helices H and I and not the underlying segment (Figures 4G–4I). It appears to adopt a shape with two unequal lobes and is larger in volume (∼60% of native MDH monomer) and more extended than the substrate density in classes A and B, contacting four GroEL subunits rather than three. This shape is somewhat reminiscent of that of a native MDH subunit (Figure 4K), suggesting that this might represent a partially folded subpopulation. In support of this idea, modestly structured folding intermediates of MDH have been shown to be recognized by GroEL (Churchich, 1998). However, because MDH is present in an ensemble of states, a specific structure cannot be identified.

Classes D and E show very small amounts of substrate density (Figure 3), but they classify into distinct groups. They appear to contain mixtures of states that are not represented in the other classes but are not sufficiently populated to form separate, homogeneous classes. They are distinct from apo GroEL because their rings of apical domains show greater deviations from 7-fold symmetry (compare top and bottom views in Figures 4D and 4E with Figures S4C and S4D).

### Disulfide Crosslinking Confirms that Nonnative Substrate Molecules Extend into the Entire Central Cavity of a GroEL Ring

**Figure 5:**
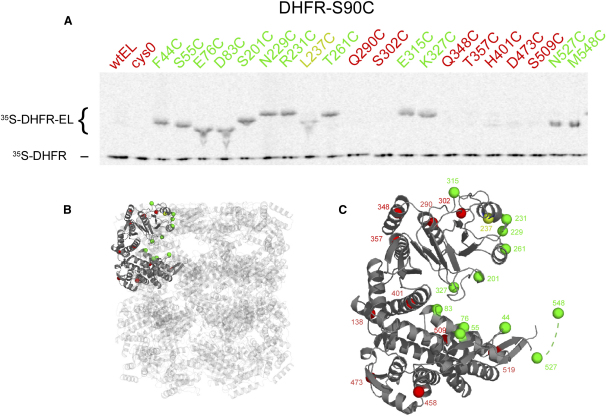
Sites on GroEL Contacted by a Bound Substrate Protein This was determined by measuring the ability of a DHFR substrate protein containing a single cysteine to become disulfide crosslinked to various GroELs bearing a single cysteine. (A) PhosphorImager analysis showing adducts formed between input ^35^S-labeled DHFR-S90C (bottom) and variant GroELs bearing a single cysteine at the positions indicated within each subunit. Red, no adduct formed. Green, adduct observed. (B and C) Positions on GroEL that exhibited crosslinking, green, or not, red, mapped onto a GroEL subunit, in the context of intact GroEL (B) and showing the subunit in isolation (C). Dotted green line in (C) designates a C-terminal segment that is not crystallographically resolvable.

The observed densities suggest that nonnative substrate protein is bound by the cavity-facing elements of the apical domains, helices H and I and the underlying segment, but can also extend into the equatorial region of the central cavity. To independently assess which surfaces can contact a GroEL-bound nonnative polypeptide, we carried out a crosslinking experiment (Figure 5). We assessed the ability of a bound substrate protein, human DHFR, containing a single cysteine, to form disulfide crosslinks with single cysteines (seven per ring) placed in a variety of surface positions both inside and outside the central cavity in a GroEL variant, Cys0, in which the three endogenous cysteines of WT GroEL have been innocuously changed to alanine (Rye et al., 1999). Twenty individual cysteine-substituted mutants were constructed, and each was shown to be fully functional both in vivo and in vitro. The DHFR was a variant containing substitutions of Cys6 to alanine and Ser90 to cysteine. This molecule exhibited the same refolding kinetics as WT DHFR and the same activity in the native state. It was preferred over WT for this experiment because position 90 lies in a region that is structured in the native state, whereas position 6 lies near the unstructured N terminus of DHFR. ^35^S-methionine-radiolabeled human DHFR was unfolded in 6 M guanidine HCl and diluted into aqueous buffer with each variant GroEL. The binary complexes, prepared in the presence of 1 mM TCEP, were then oxidized with diamide, alkylated with iodoacetamide, denatured in nonreducing sample buffer, and separated in SDS-PAGE, followed by PhosphorImager analysis. Crosslinking was detected by conversion of the input 21 kDa radiolabeled DHFR species to a radiolabeled GroEL-DHFR adduct of ∼79 kDa (Figure 5). In control tests, Cys0 did not form an adduct, lacking a cysteine to which DHFR could crosslink (Figure 5A, lane 2). Similarly, WT GroEL did not form an adduct, consistent with the lack of availability of its three natural cysteines for crosslinking with a protein bound in the central cavity (Figure 5A, lane 1). In particular, the natural cysteines, Cys138, Cys458, and Cys519, are positioned at the outside aspect of the intermediate domain (Cys138) and buried in the equatorial domain (Cys458 and Cys519).

In contrast with Cys0 and WT GroEL, all of the cysteine variants lining the central cavity at both apical and equatorial levels formed readily observable crosslinks with the bound DHFR. In the apical domain, residues 229 and 231 on helix H, 261 on helix I, and 201 on the underlying extended segment, all immediately adjoining hydrophobic residues involved in polypeptide binding, formed contacts with the DHFR. Residue 237 on helix H, a leucine normally involved in polypeptide binding, also formed a crosslink, albeit somewhat less efficiently, when substituted with cysteine. Residue 327, lying on a cavity-exposed loop between two strands forming the core of the apical domain, also formed a crosslink. At the equatorial level, residues lying on top of the domain, 44, 55, 76, and 83, all formed crosslinks. In addition, residues of the flexible C-terminal tails, 527 at the proximal end and 548 at the very C terminus, formed crosslinks. Thus, all positions that were tested inside the central cavity were accessible to nonnative polypeptide. In addition, position 315 on the top surface of the subunit, lying on a flexible segment immediately behind helix H, formed a crosslink. This implies that the middle portion of at least some DHFR molecules can extend out of the cavity onto the top surface of the apical domain. Notably, however, positions on the top surface of GroEL that are radially further out from the cavity, e.g., aa 290 and 348, were not crosslinked. Virtually identical results were obtained with the larger substrate protein, Rubisco, where five cysteines were available for crosslinking (see Figure S3). We conclude, in agreement with the cryo-EM data, that, while nonnative proteins may be bound by the apical domains, they can explore the entire chaperonin cavity and can extend outside of it over the nearby top surface of the domains.

### Domain Movements in GroEL Induced by Substrate Binding

**Figure 6:**
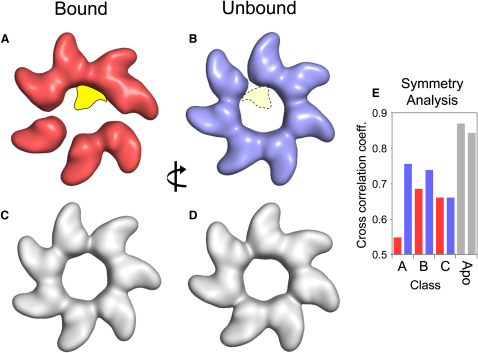
Disruption of GroEL Ring Symmetry in the Presence of Bound Substrate (A and B) Surface representation of the apical rings created by fitting the individual domains to the class A cryo-EM structure, converting to electron density, and low-pass filtering to 20 Å resolution. Shown in (A) are the apical domains of the substrate-bound ring along with an outline of the denatured MDH location in this ring (yellow). Shown in (B) are apical domains of the unbound ring. The dotted outline marks the location of the substrate on the opposite (bound) ring as seen from this view. The views in (A) and (B) are related to each other by a 180° rotation around a vertical axis as indicated by the arrow. (C and D) Shown for comparison are the two apical ring densities created by fitting the individual domains to the asymmetric EM map of apo GroEL(D473C) (Figure S4) and low-pass filtering to 20 Å. The bound apical ring of the class A structure (A) shows striking asymmetry compared to the apo GroEL rings (C and D), with four apical domains bunching around the substrate and gaps forming at the opposite side of the ring. Notably, a small gap was also formed in the unbound ring of the class A structure (B) between two apical domains aligned with the substrate position. (E) Symmetry analysis of apical rings in the three bound classes and in the apo GroEL asymmetric structure. The crosscorrelation coefficients represent the degree of 7-fold symmetry in each apical ring. Red columns represent substrate-bound rings in classes A–C, blue columns represent the unbound rings, and gray columns represent the two rings of the asymmetric apo GroEL. The substrate-bound rings of classes A-C feature the least symmetry, whereas both apo GroEL rings are considerably more symmetric.

Does GroEL bind substrates passively within a ring, or do the apical domains adjust in response to binding as suggested by earlier studies (Chen and Sigler, 1999)? To address this question, we examined the positioning of the apical domains relative to those of apo GroEL. For this comparison, we calculated an asymmetric reconstruction from the control images of apo GroEL(D473C) (Figure S4). Shown in Figure 6 are comparisons of the fitted apical domains for class A (Figures 6A and 6B), which has the best resolution of the three classes containing significant substrate density, with those of apo GroEL (Figures 6C and 6D). In general, the apical domains seem to be bunched closer together around positions of substrate contact, leaving large gaps between neighboring apical domains in the other part of the ring. Disruption of the apical ring symmetry is seen in all five classes, but not in the asymmetric reconstruction of apo GroEL. The graph in Figure 6E quantifies the reduced degree of 7-fold symmetry in the apical rings with bound substrate compared to the higher symmetry seen in the rings of apical domains in apo GroEL.

## Discussion

The cryo-EM structures presented here provide unprecedented, direct observations of the distribution of a full-length, nonnative polypeptide substrate bound in the cavity of an open GroEL ring. Despite the disordered structure of bound substrate, as documented previously by hydrogen exchange and NMR studies, and its small size relative to the whole complex, the use of single-particle EM enabled the visualization of subpopulations of nonnative substrate localized to specific regions on the chaperonin cavity surface. This was achieved by applying statistical analysis to sort the images into homogeneous subsets for 3D reconstruction without imposing symmetry.

### Nonnative Substrate Density Visualized in the EM Maps

Significant additional density appeared in three image classes, extending from the cavity-facing aspect of one GroEL ring. These additional densities can be explained only by the presence of nonnative MDH, since all GroEL domains can be accounted for by the remaining density with no substantial modification of their structures. Identification of substrate contributions to the images is also supported by the eigenimage analysis showing significant variations in density inside the GroEL cavity, independent of variations in orientation and which, most importantly, are not present in apo GroEL data sets. Classes A–C present substrate topologies that were sufficiently populated in the data set to reveal anywhere from 25% to 60% of the volume of the substrate, the bound MDH subunit, as occupied in its native state. It appears that the greater the binding surface on GroEL occupied by MDH, the more substrate volume is visible in the maps, presumably because the substrate is more restricted by multiple contacts, e.g., in class C compared to class A.

### Binding of Substrate to Multiple Apical Domains and Effect of Binding on Structure of the Ring

The density of the bound MDH is seen adjacent to three or four consecutive apical domains. Such multivalent binding agrees with an earlier genetic and biochemical study observing simultaneous binding of nonnative protein to multiple apical domains (Farr et al., 2000). In particular, MDH was efficiently bound by GroEL only when at least three consecutive apical domains were binding proficient. Here, we observed substrate density abutting three consecutive domains, although size considerations predict that additional apical domains could have also interacted. The cavity diameter in apo GroEL is 45 Å, whereas a monomer of native MDH is ∼57 Å long and ∼38 Å wide. This indicates that nonnative MDH is large enough to simultaneously bind all of the apical domains of an open ring. However, even in a nonnative, expanded state, the MDH density appears to be restricted to only one side of the GroEL central cavity, and the remainder of the visible density in classes A and B extends deeper into the cavity rather than interacting with hydrophobic binding sites on the opposite apical domains. Therefore, the interaction of MDH with GroEL must cause changes in the conformation of GroEL and/or of nonnative MDH that disfavor binding to the remaining apical domains. The observed bunching together of substrate-bound apical domains and the alteration of the remaining binding surface of the ring by separation of adjacent apical domains may explain why MDH binds mainly to three of the available sites in classes A and B and four in class C.

These apical domain movements are the main effects of substrate binding on GroEL conformation observed in our study. Falke et al. (2005) described minor rotations of the apical domains in the GroEL-glutamine synthetase structure that were much smaller than the domain movements reported here. The discrepancy could be the result of differences in binding mode of the two substrates, which are dissimilar in size and structural features, but more likely is that the movements observed here were obscured by 7-fold averaging of the GroEL-glutamine synthetase structure.

### Substrate Localization and Implications for the Encapsulation Mechanism

Although the original mutational studies identified the helix H/I surface and underlying segment as essential hydrophobic binding sites (Fenton et al., 1994), detailed structural studies have so far only shown short peptides binding in the groove between helices H and I (Buckle et al., 1997; Chen and Sigler, 1999; Kobayashi et al., 1999). Here we have observed populations of polypeptide substrate molecules variously anchored to the cavity wall via the originally identified hydrophobic surfaces of the apical domains, but with a preference for association with helix I, situated relatively deeply in the central cavity. In addition, density could be observed extending down as far as the equatorial region, potentially interacting with the flexible C-terminal tails (Ojha et al., 2005; Tang et al., 2006). Correspondingly, the disulfide crosslinking study indicated that polypeptide can populate any position inside the central cavity, including the equatorial zone.

The preferential location of substrate adjacent to the lower aspect of the binding surface potentially leaves room for the GroES mobile loop to bind above it in the groove between helices H and I as seen in the GroEL-GroES complex (Xu et al., 1997). This topology makes it easier to understand encapsulation by GroES without polypeptide escape (Cliff et al., 2006). It is harder, however, to understand how encapsulation might proceed with class C, where polypeptide is bound at the inlet to the cavity. This would obstruct GroES binding to the H-I groove on the four occupied subunits. In this case, encapsulation might proceed by GroES binding first to the unoccupied apical domains.

In summary, this work has provided a 3D description of the distribution of nonnative polypeptides in the chaperonin cavity at the first stage in the process of chaperonin-assisted protein folding.

## Experimental Procedures

### Proteins and Reagents

#### 6-His Modification of GroEL D473C

One μmol of a histidine hexapeptide in 2 ml of 25 mM KP (pH 8) in 50% ethanol was first modified with 10 μmol of the heterobifunctional crosslinker Sulfo SMCC (Pierce) at 20°C for 60 min, then quenched by the addition of 100 μmol glycine. This modified peptide (∼0.2 μmol) was then added to 0.1 μmol of GroEL D473C in 8 ml of 50 mM HEPES (pH 7.4), 50 mM KCl, and 1 mM TCEP. After 12 hr, the reaction was quenched with 0.8 ml of 0.25 M reduced glutathione. Histidine-modified GroEL was then purified using Talon resin (BD Biosciences). Porcine mitochondrial MDH was purchased from Roche UK.

### Binary Complex Preparation

MDH (25 μM) was denatured in 6 M urea, 50 mM Tris (pH 7.5), and 10 mM DTT for 30 min. It was then rapidly diluted 100-fold into a solution of 0.1 μM GroEL oligomers in 50 mM Tris (pH 7.5), 50 mM KCl, 10 mM MgCl_2_, and 1 mM DTT such that MDH was at 2.5 molar excess over GroEL oligomers. The mixture was incubated for 10 min at 24°C, centrifuged for 10 min to remove aggregates, and then diluted to final MDH and GroEL concentrations of 0.125 μM and 0.05 μM, respectively, leaving 30 mM of residual urea.

### EM Data Collection and Preprocessing

The complexes were vitrified on a thin, continuous carbon film supported by a layer of holey carbon film. Images were recorded on Kodak S0-163 film (Sigma UK) at a magnification of 50,000 using an F20 FEG at 200 kV (FEI Eindhoven, The Netherlands) and a Gatan cryotransfer stage maintained at −170°C. A subset of the images was recorded using an F30 FEG at 200 kV and a magnification of 39,000. Imaging was done using a defocus range of 0.7–3 μm and an electron dose of 10–20 e^−^/Å^2^. The films were digitized at a step size of 7 μM using a Zeiss Scai scanner, giving either 1.4 Å or 1.8 Å per pixel.

The contrast transfer function (CTF) was determined for each image using the MRC program CTFFIND3 (Mindell and Grigorieff, 2003). Micrographs with noticeable drift or astigmatism were discarded. Approximately 40,000 particles were selected interactively using the MRC program XIMDISP (Crowther et al., 1996), extracted into boxes of 512 × 512 pixels using the MRC program LABEL, and phase corrected using SPIDER (Frank et al., 1996). After CTF correction, the box size was cropped and sampling reduced so that all images were at 2.8 Å per pixel in 160 × 160 pixel boxes. Images were band-pass filtered between 200 Å and 3 Å and normalized to zero mean and the same sigma in SPIDER.

### Classification and 3D Reconstruction

Image processing was done in SPIDER (Frank et al., 1996) and IMAGIC (van Heel et al., 1996) programs. Images were centered, subjected to MSA, and classified with approximately ten images per class. After excluding obviously defective images, a total of 34,761 side-view and several hundred end-view images were retained for subsequent processing.

We began by reconstructing a 7-fold symmetric map from 8000 images out of an initial data set of 9500 (Figures 1B–1D). The initial map was calculated from class averages by angular reconstitution (van Heel et al., 2000) and then refined using projection matching (Penczek et al., 2006) with an initial angular increment of 6° until there was no significant change in angle assignments between successive rounds.

In order to begin asymmetric reconstruction, several different asymmetric starting models were created by removing different portions of the substrate density from the cavity in the upper ring of the symmetrized map. The choice of asymmetric starting model did not significantly affect the final results. The strategy used for sorting the data set and 3D reconstruction is summarized in the flow chart in Figure S5. At each stage, 3D maps were separately refined by projection matching and then assessed for homogeneity of their constituent images by MSA. If the eigenimages showed evidence of structural heterogeneity, the images were divided into subclasses and separate 3D maps reconstructed. Sorting of the images into different structural classes was refined by competitive projection matching, in which all 40,000 images in the data set were compared to reprojections of all the current maps and assigned to the structure with which they showed the highest crosscorrelation. A crosscorrelation threshold was applied after each competitive projection matching, selecting ∼85% of the images for that round. In order to base the first few rounds of competitive alignments only on substrate density and not on asymmetries in GroEL, the GroEL part of the map was symmetrized and the substrate was left asymmetric. In addition, density was thresholded to truncate extreme values. The procedure of image separation was iterated four times with refined models and decreasing angular steps down to 1°. Ultimately, the data set was subdivided into five structural classes yielding five different 3D maps. Notably, although substrate density in the substrate-occupied map at an early stage of refinement was bound to apical domains on opposite sides of the ring (Figure S6), in the final refined maps it was found bound almost exclusively to contiguous apical domains, indicating an absence of reference bias.

After the data set was divided into the final five classes, the vast majority of images did not change in their assigned angles and class, and MSA did not show evidence of intraclass structural heterogeneity (Figure S2). All attempts to classify into fewer groups—for example, classifying without class D (which does not show clear substrate density)—resulted in reduction of overall stability of classes. Attempts to further split any of the classes did not result in any significantly different structure. Maps were loosely masked to exclude noise more than 5 pixels outside the map surface.

### Atomic Structure Fitting and Determination of Difference Densities

For fitting the symmetrized map, one GroEL subunit from each ring of the crystal structure was split into the three separate domains, equatorial, intermediate, and apical. These domains were then fitted as six rigid bodies into the cryo-EM density map using the program URO (Navaza et al., 2002), with 7-fold symmetry. For the five asymmetric maps, individual domains were docked separately in each of the 14 GroEL subunits with URO as 42 independent rigid bodies. Manual adjustments to the URO fit were necessary for some intermediate domains, which are small and flexible, and for the apical domains of the substrate-bound ring of classes B and C, where the substrate forms a continuous density with a large part of the binding surface. These adjustments were made only when the URO fit did not place the domain inside the density, or the hinge regions of adjacent domains were not in close proximity.

To approximate the isolated substrate density in the central cavity, the following procedure was applied. Difference densities were calculated between the cryo-EM structures of classes A–C, low-pass filtered to 14 Å, and corresponding fitted GroEL atomic coordinates, which were converted to density maps and filtered similarly. All maps were normalized and binarized at a threshold level representing the full molecular mass, followed by subtraction of the fitted GroEL density from the maps of the complexes. The major positive difference density was found in the GroEL central cavity. Significant positive difference appeared also in the C-terminal region, especially in class C.

### Apo GroEL(D473C) Data Collection and Image Processing

Cryo-EM data collection and digitization of apo GroEL(D473C) were done similarly to those for the GroEL-MDH complex (images recorded only on Tecnai F20 at 50,000 magnification), except that the sample was imaged in an unsupported vitreous ice layer. From a data set of 10,400 particles, a total of 8,810 side-view and several hundred end-view images were retained for subsequent processing, after excluding defective images. A 7-fold symmetric starting model produced by angular reconstitution was used as a starting model for the asymmetric map, which was refined by projection matching.

### Disulfide Crosslinking of Binary Complexes of DHFR and GroEL Cysteine Variants

^35^S-labeled DHFR was unfolded by diluting a 300 μM stock solution 10-fold into 7 M guanidine HCl, 100 mM HEPES (pH 7.4), and 10 mM TCEP. Binary complexes with GroEL cysteine variants were formed by diluting the unfolded DHFR 100-fold into a buffer (50 mM Bis Tris [pH 6.0], 50 mM KCl, 10 mM MgCl_2_, 1 mM TCEP) containing 1 μM chaperonin. After 5 min at 20°C, the sample was centrifuged at 17,000 × g for 5 min to remove any precipitated DHFR. Disulfide crosslinking was initiated by adding diamide to a final concentration of 2 mM. After 15 min, free cysteines were blocked by the addition of 10 mM iodoacetamide. The sample was then separated in nonreducing 6% SDS-PAGE, and crosslinked radiolabeled products were visualized by PhosphorImager analysis. All of the crosslinks observed at 10 min were also observed when crosslinking was carried out for 30 s.

## Figures and Tables

**Table 1:** Experimental Details

Structure	Sample	Symmetry	Number of Images in Structure	Resolution (Å)
Initial GroEL-MDH	GroEL(D473C-6His)-MDH	7-fold	8000	8.7
GroEL-MDH class A	GroEL(D473C-6His)-MDH	no	5800	10.6
GroEL-MDH class B	GroEL(D473C-6His)-MDH	no	5000	10.7
GroEL-MDH class C	GroEL(D473C-6His)-MDH	no	3800	11.2
GroEL-MDH class D	GroEL(D473C-6His)-MDH	no	7000	10.5
GroEL-MDH class E	GroEL(D473C-6His)-MDH	no	8800	9.7
Apo GroEL symmetric	GroEL(D473C)	7-fold	6700	8.7
Apo GroEL asymmetric	GroEL(D473C)	no	6800	10.0

## Supplemental Data

Documents S1. Six Figures
