# Supplementary material for: Topologies of a Substrate Protein Bound to the Chaperonin GroEL
Source: Mol Cell. 2007 May 11;26(3):415–26. doi: 10.1016/j.molcel.2007.04.004 (PMC1885994; doi:10.1016/j.molcel.2007.04.004)
Supplement: Documents S1. Six Figures [file mmc1.pdf]

Molecular Cell, Volume *26*

Supplemental Data

Topologies of a Substrate Protein Bound to the Chaperonin GroEL

Nadav Elad, George W. Farr, Daniel K. Clare, Elena V. Orlova, Arthur L. Horwich, and Helen R. Saibil

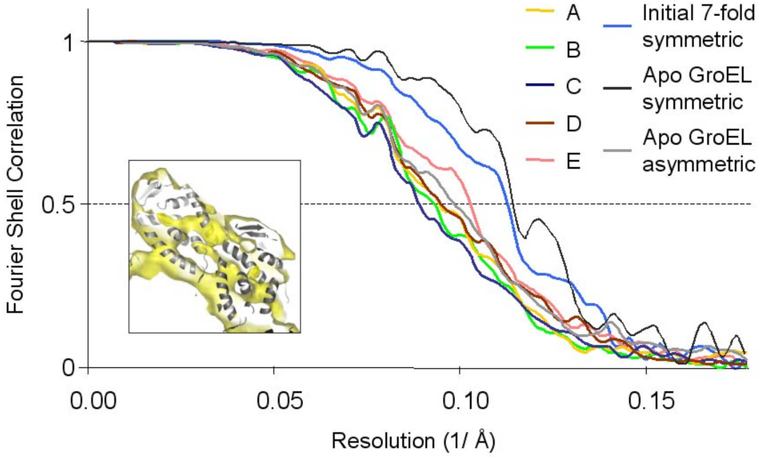

Figure S1. Resolution Curves

Fourier shell correlations (FSC) of the 5 different GroEL-MDH asymmetric cryo-EM structures made from the classified images (A-E). Also presented are the FSCs of the initial 7-fold symmetric GroEL-MDH structure (Figure 1B-D), the apo GroEL symmetric structure and the apo GroEL asymmetric structure. A section of the density map of complex A after amplitude sharpening (inset) shows the degree of definition of α-helical features expected for maps in this resolution range.

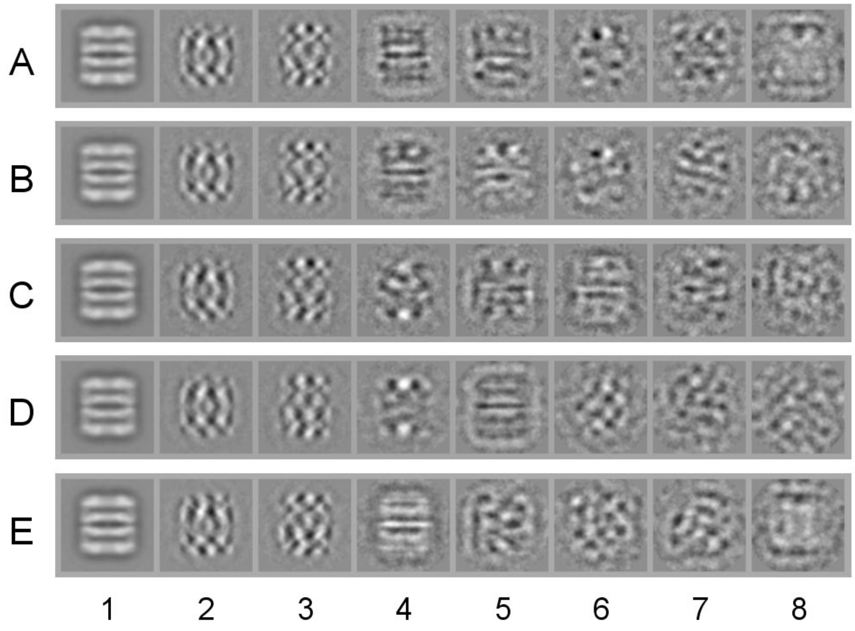

Figure S2. Intraclass Variations in the Final Five Classes

First 8 eigenimages derived from multivariate statistical analysis (MSA) within classes A-E. Using eigenimages we were able to progressively subdivide the data set into increasingly homogeneous subsets. The eigenimages indicate that this is the maximum homogeneity that can be achieved with this data set. As opposed to previous eigenimages that were calculated during the refinement procedure (e.g. the eigenimages in Figure 2A) classification based on any single eigenimage here did not result in a significant new topology. As in Figure 2A eigenimages, the first eigenimage in all classes is the total average, followed by two eigenimages that reflect the different GroEL views (2,3). However, in these MSAs, eigenimages that show strong deviations in the GroEL cavity (namely A6, B6, C4 and D4) reflect different orientations of asymmetric density around the central cavity. Classifying based on these eigenimages results in uneven distribution of Euler angles in the resulting classes, showing that they report on changes in orientation rather than changes in structure.

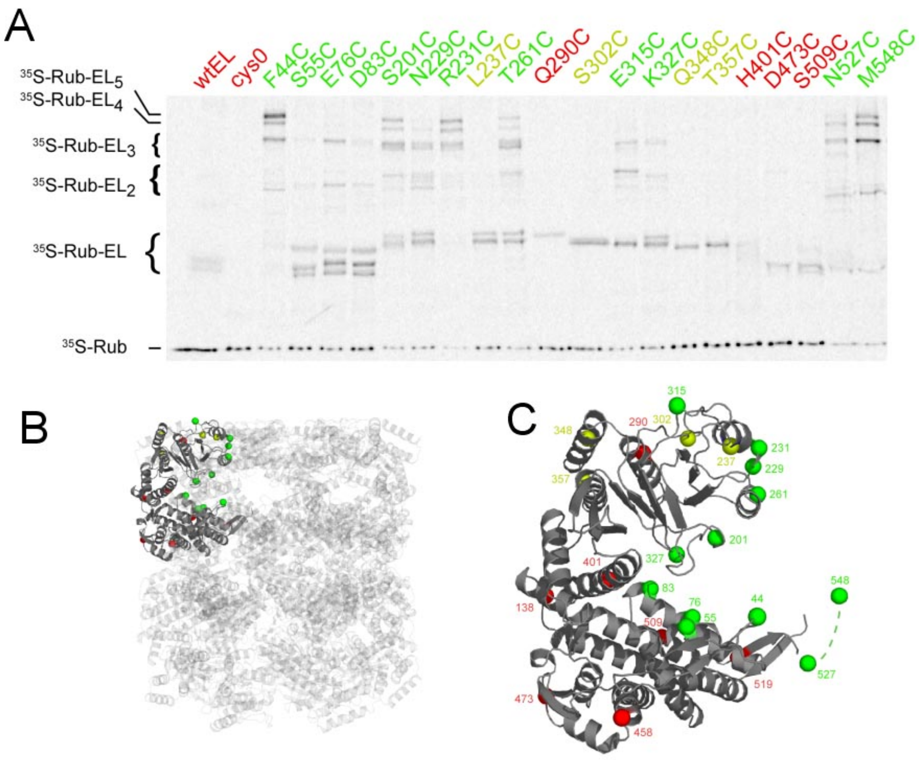

Figure S3. Sites on GroEL Contacted by Bound Rubisco

This was determined by measuring ability of a Rubisco substrate protein, containing five cysteines, to become disulfide crosslinked to various GroELs bearing a single cysteine. (A), PhosphorImager analysis showing adducts formed between input ^{35}S-labeled Rubisco (bottom) and variant GroELs bearing a single cysteine at the positions indicated within each subunit. Each rubisco can become crosslinked to up to five GroEL subunits. Red, no adduct formed. Green, adduct observed. (B,C) Positions on GroEL that exhibited crosslinking, green, or not, red, mapped onto a GroEL subunit, in the context of intact GroEL (B) and showing the subunit in isolation (C). Dotted green line in c designates C-terminal segment that is not crystallographically resolvable.

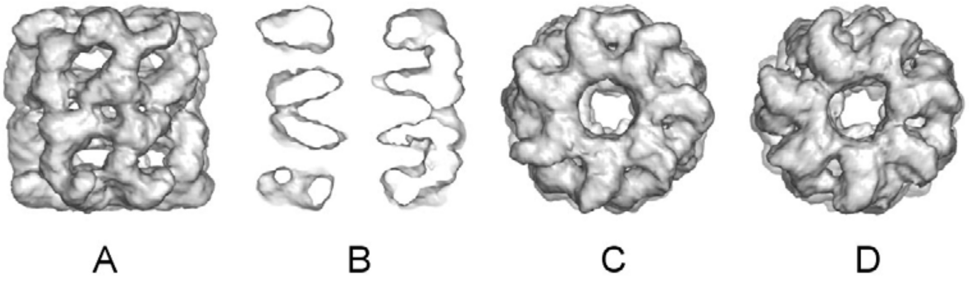

Figure S4. Apo GroEL(D473C) Asymmetric Structure

Surface representation of apo GroEL(D473C) asymmetric cryo-EM structure made from a data set of 8,800 images, out of which 6,800 images were included in the final map. (A) Side view, (B) central section, (C) top view and (D) bottom view.

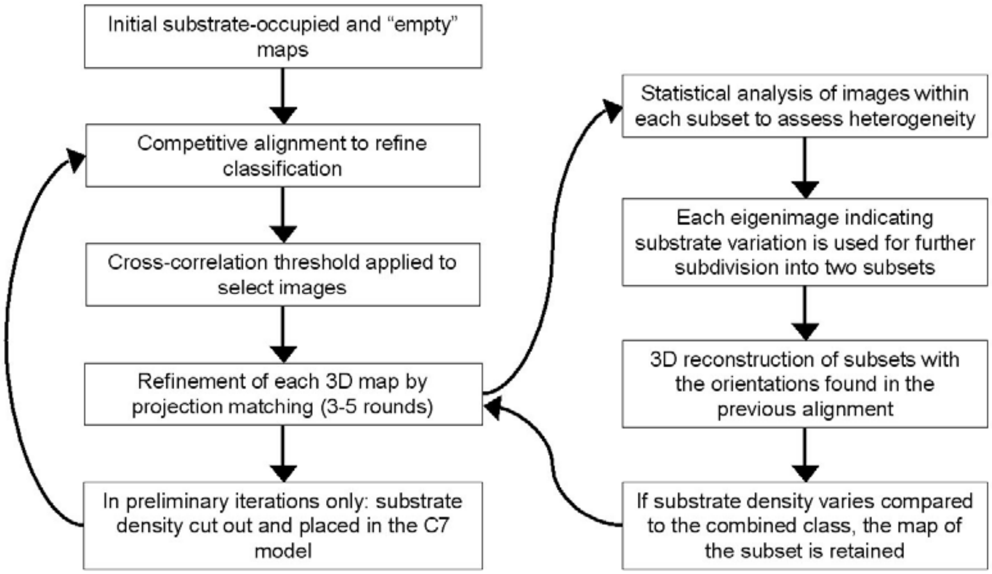

Figure S5. Flowchart for the Main Classification Procedure

Flowchart describing the main classification and refinement procedure starting from the initial substrate-containing map, along with a second map (“empty”) from which substrate density was removed.

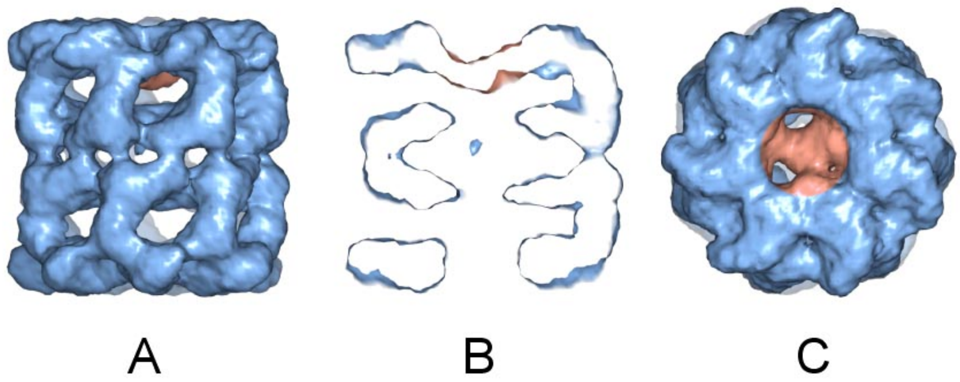

Figure S6. Starting Model for Classification of Substrate Complexes

Surface representation of the substrate-occupied map used to start the main classification procedure. The reference was made by extracting the central density from a previously calculated GroEL-MDH asymmetric map (as described in the method section) and placing it in the initial 7-fold symmetrised GroEL-MDH map (Figure 1B-D), replacing the substrate density there. Notably, in this initial model the extra density (orange) bridges from one side of the GroEL cavity to the other, a topology that is not seen in any of the final 5 maps, indicating an absence of reference bias.

The starting substrate-free map was made from the same initial 7-fold symmetrised GroEL-MDH map (Figure 1B-D), but the central density was masked out. Similar results were obtained with different models used at this and other stages of the analysis.
